# Supplementary figures and images for: Regulation of gliotoxin biosynthesis and protection in Aspergillus species
Source: PLoS Genet. 2022 Jan 18;18(1):e1009965. doi: 10.1371/journal.pgen.1009965 (PMC8797188; doi:10.1371/journal.pgen.1009965)

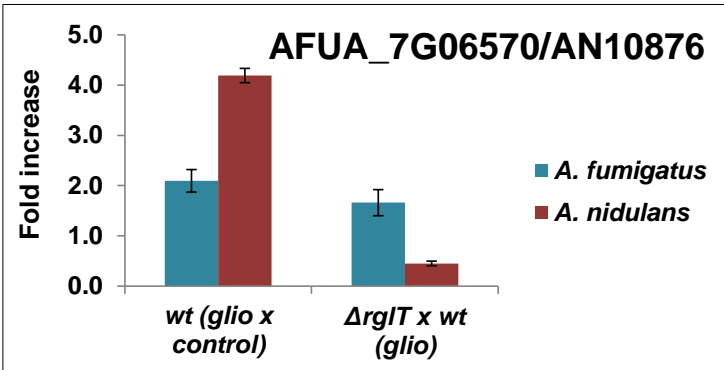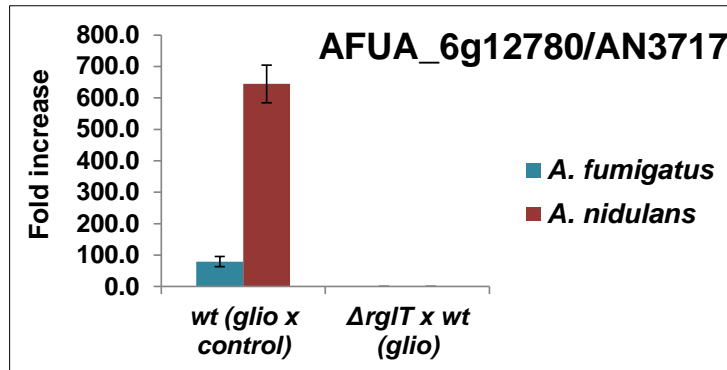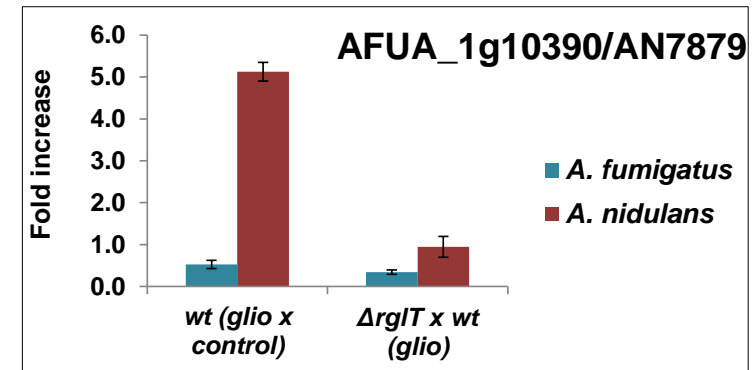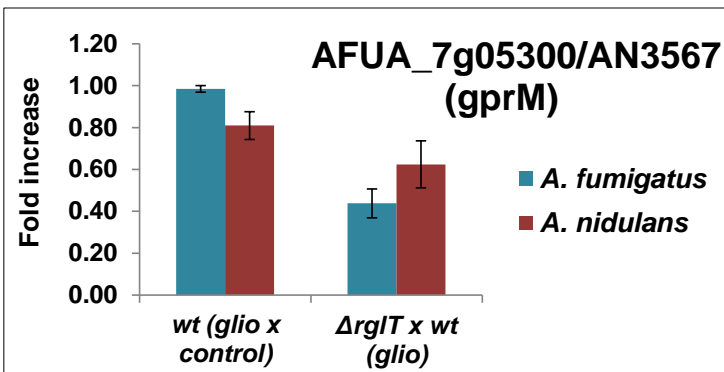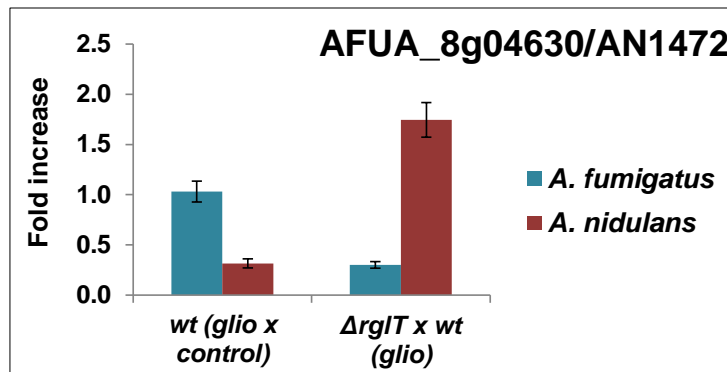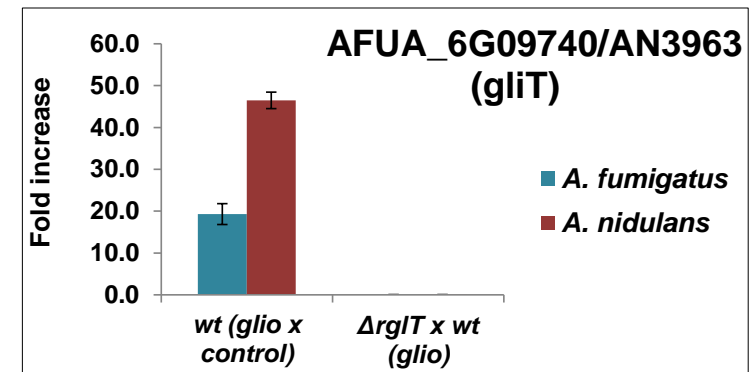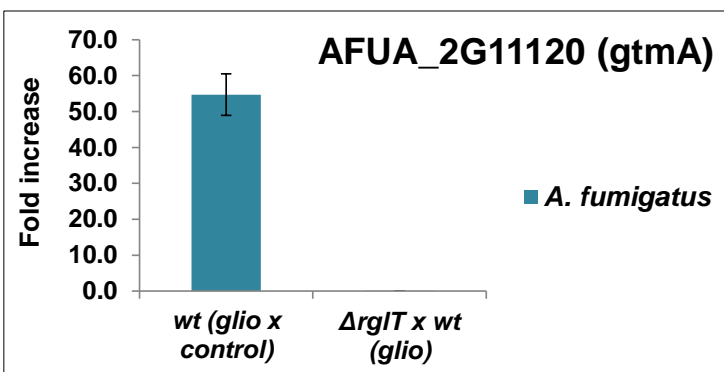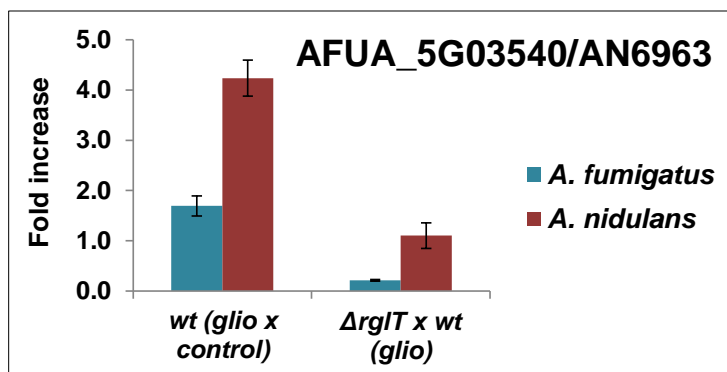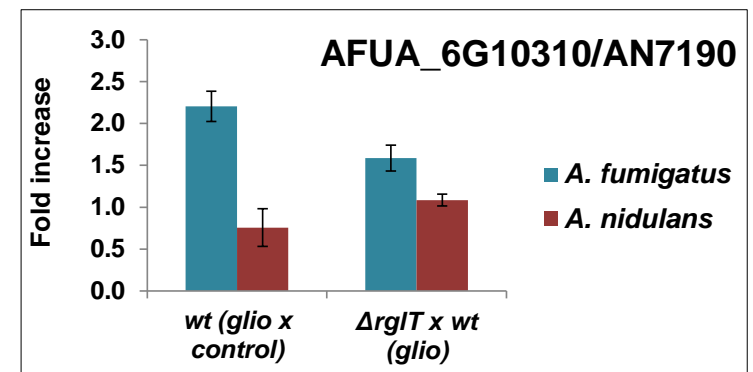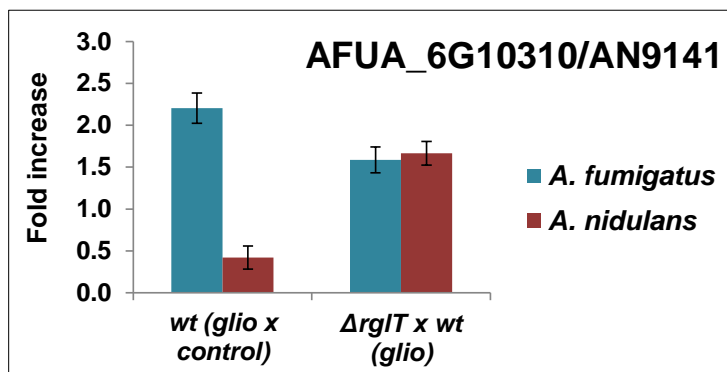

Supplement: S1 Fig — (PDF) [file pgen.1009965.s001.pdf]

Control (+SO<sub>4</sub>)

+ Met 1mM

+ Cys 1mM

- S

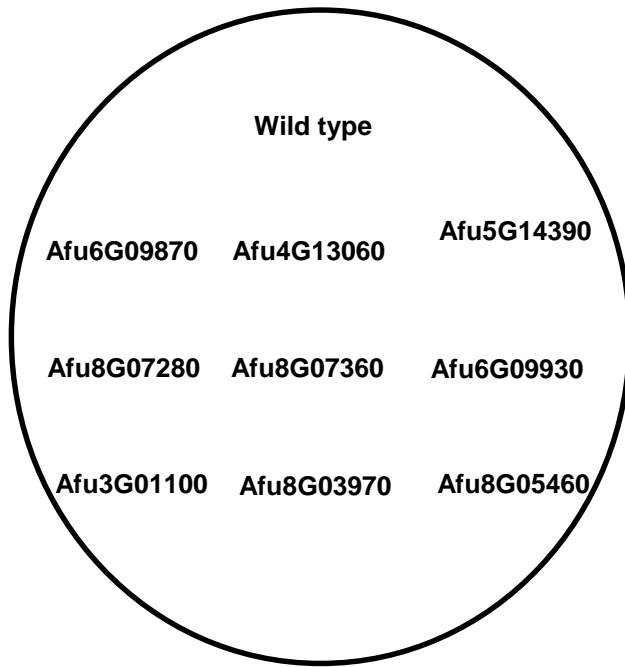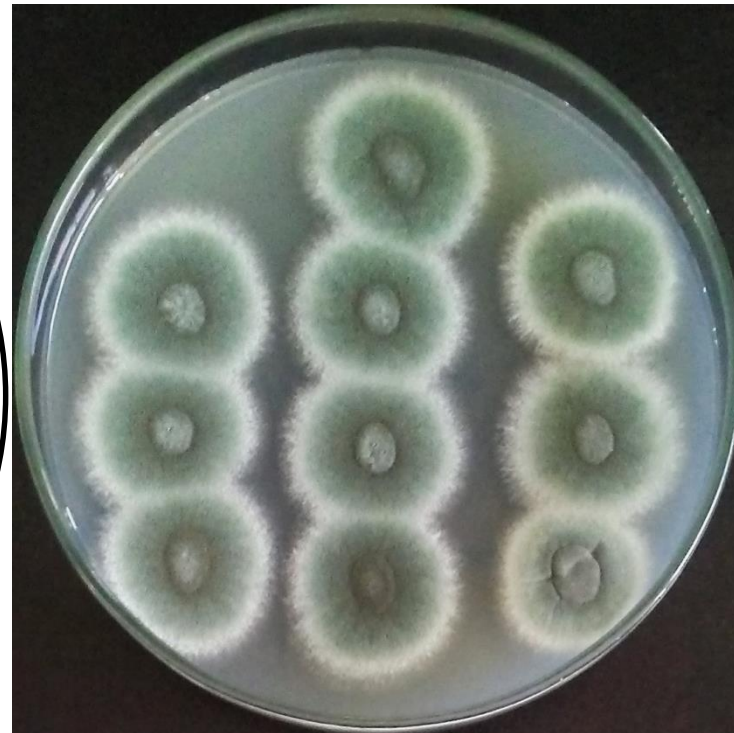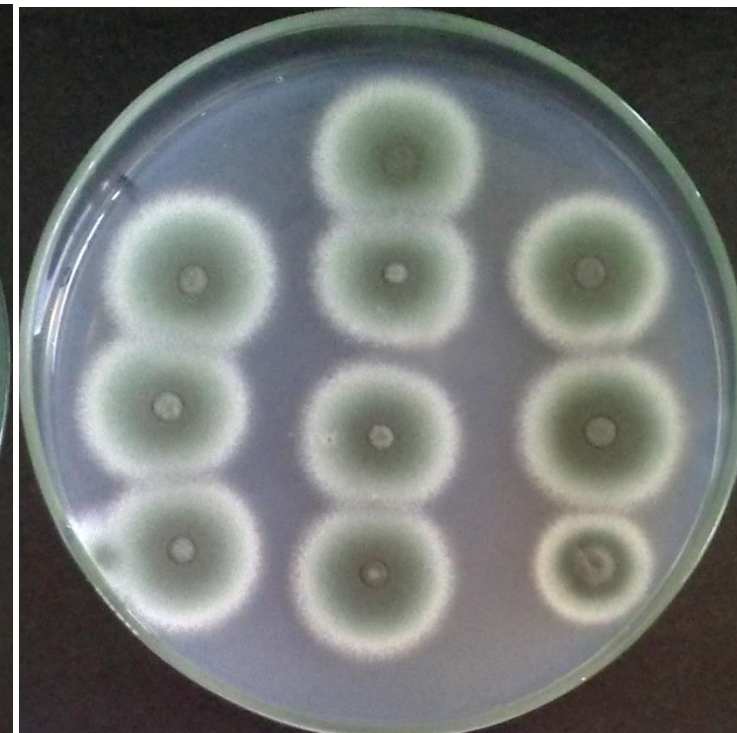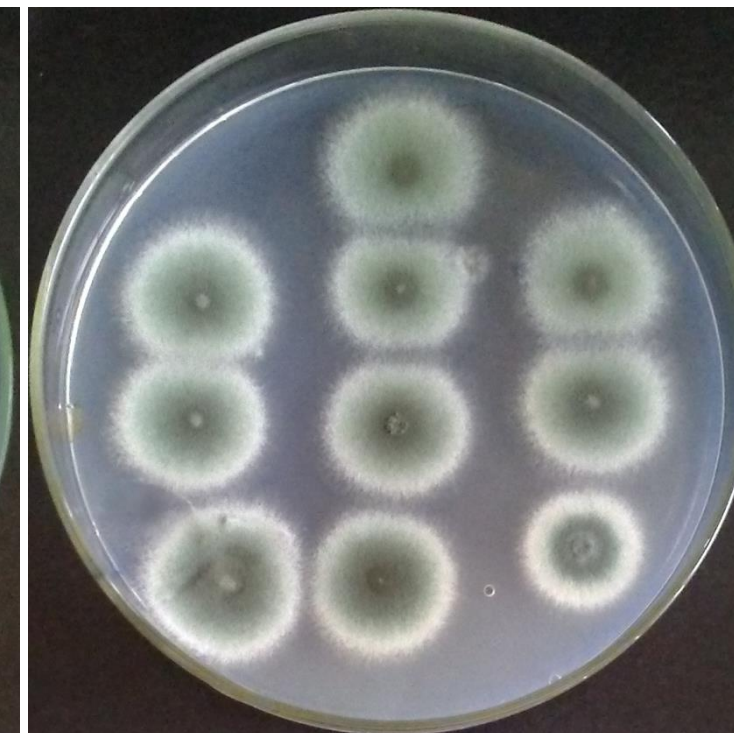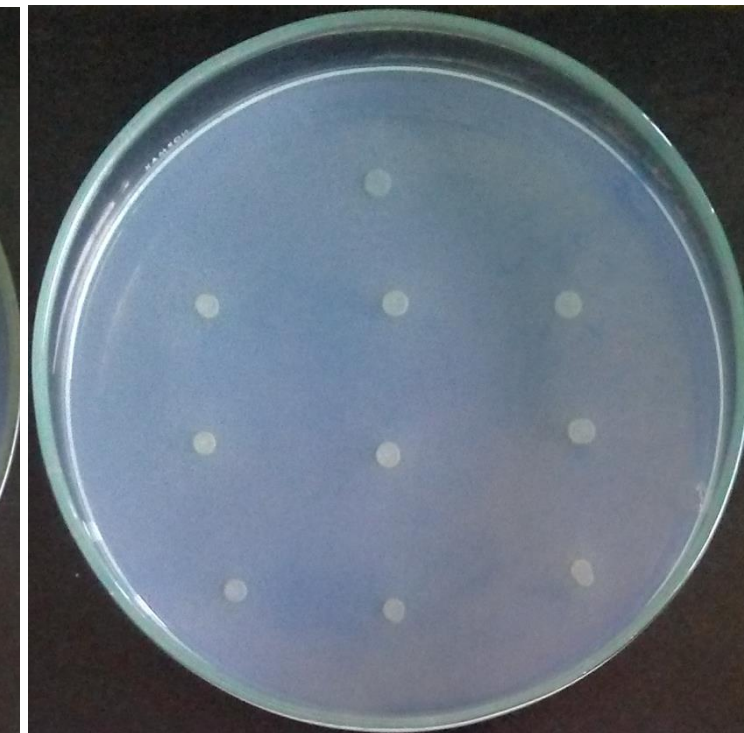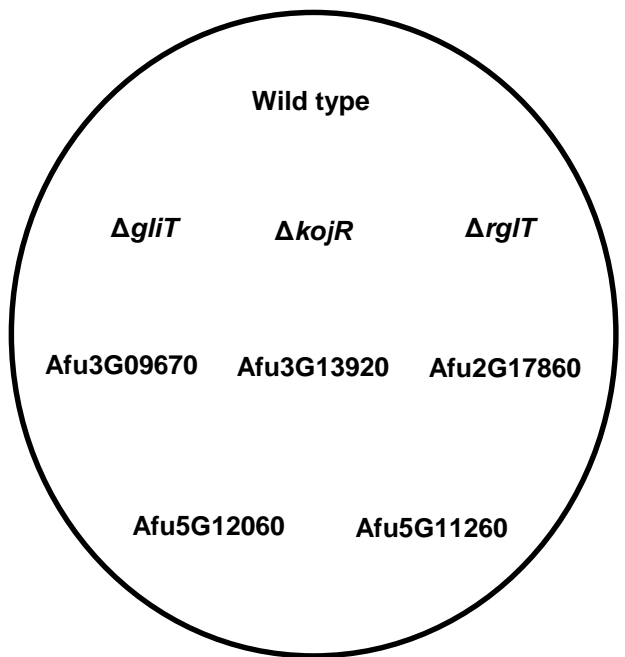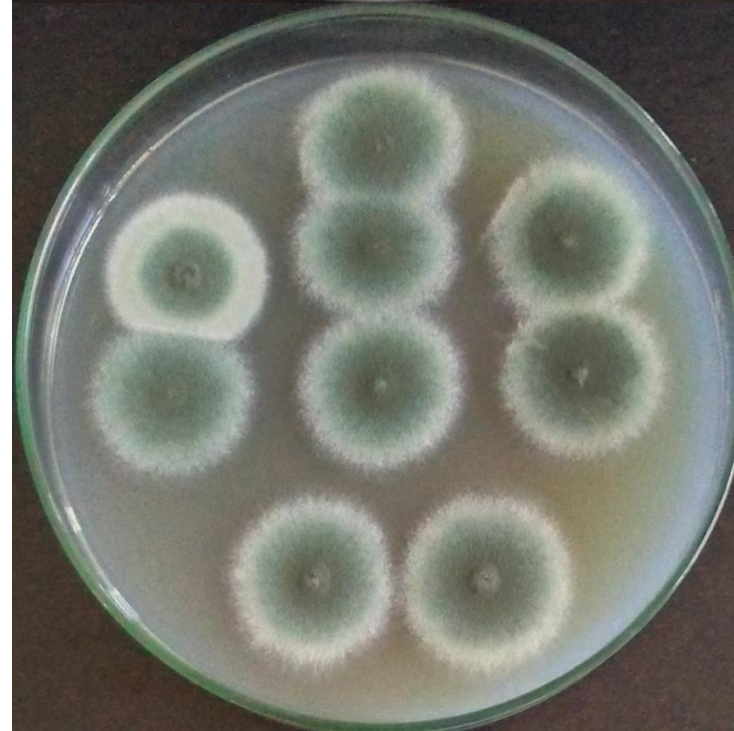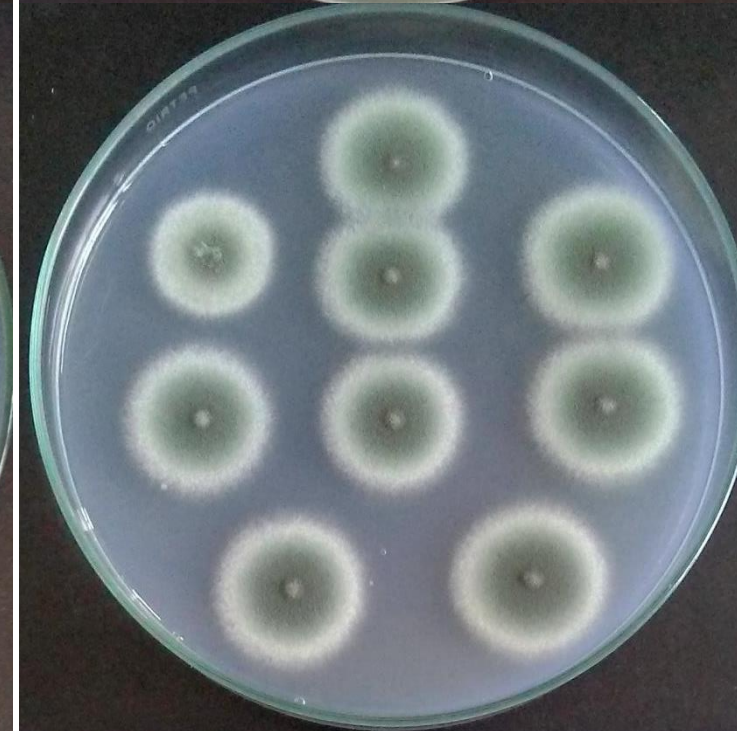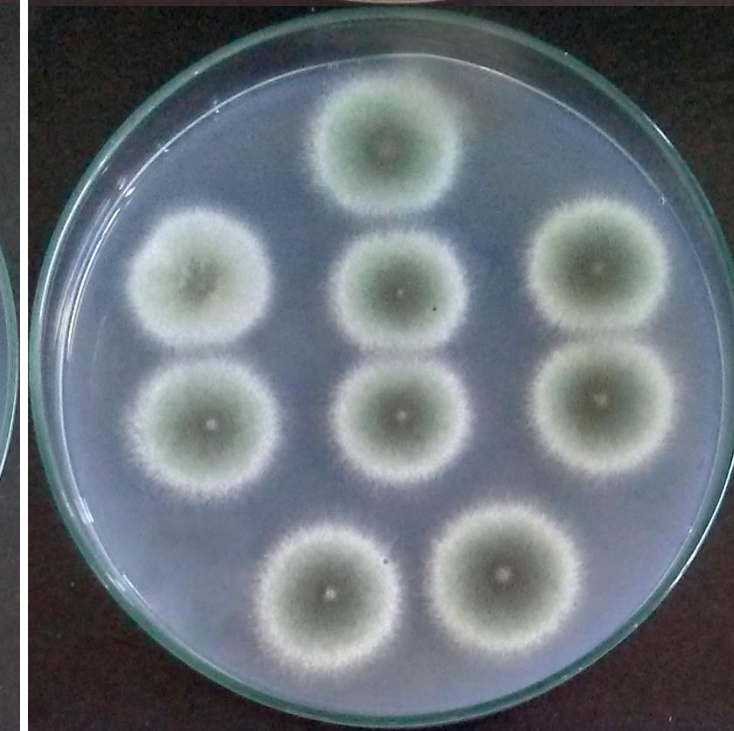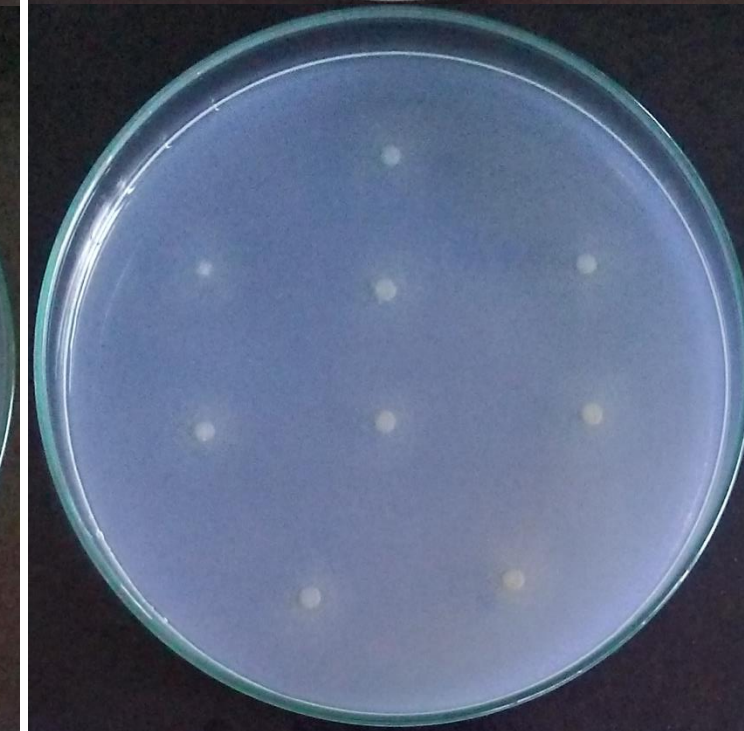

Supplement: S3 Fig — (PDF) [file pgen.1009965.s003.pdf]

A.

### Gliotoxin

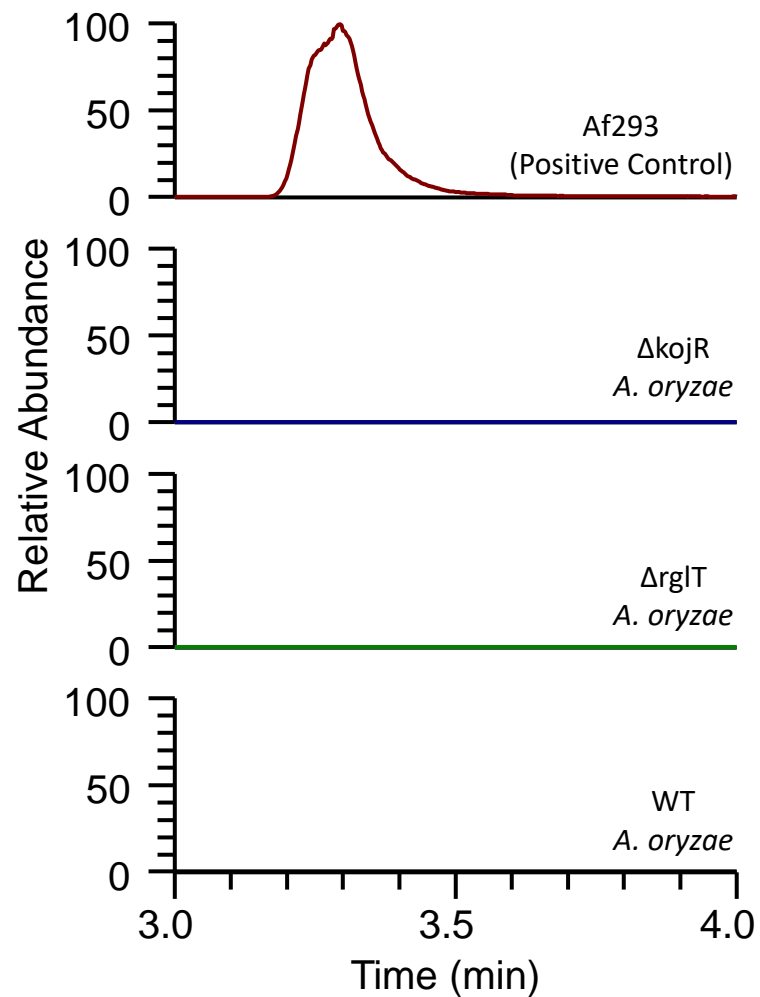

B.

### Bis(methylthio)gliotoxin

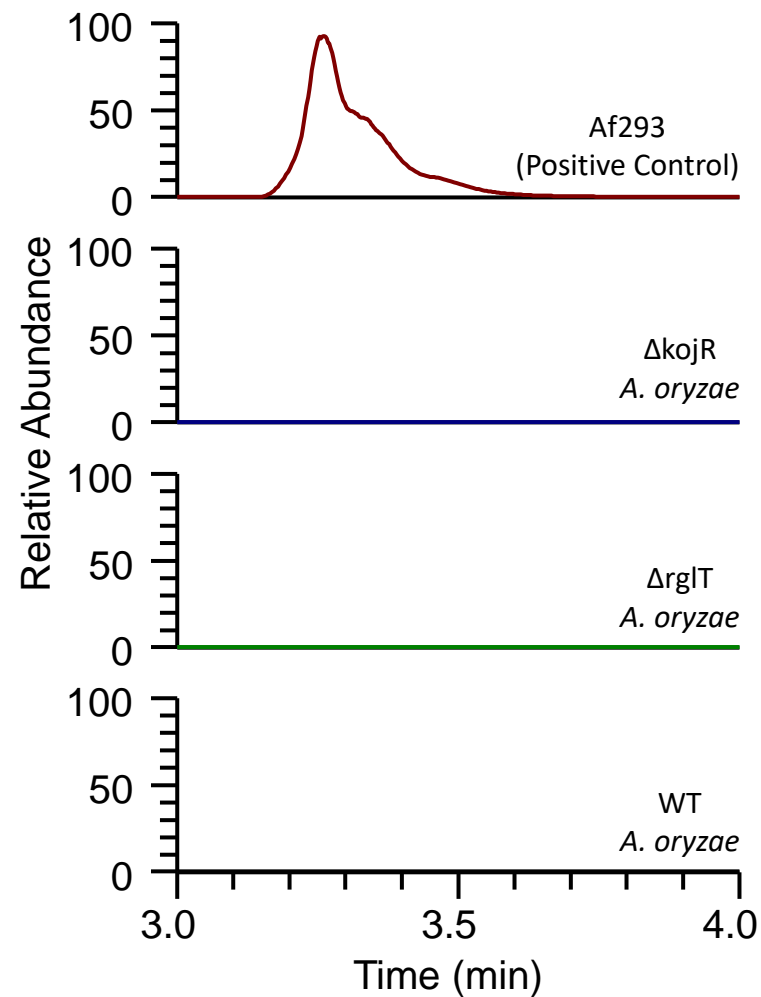

Supplement: S4 Fig — Results show that the A. oryzae WT and deletion strains do not produce (A) gliotoxin or (B) bis(methylthio)gliotoxin. The data are presented as extracted ion chromatograms (XIC) from selected ion monitoring (SIM) using the protonated mass of gliotoxin (C13H15N2O4S2; [M+H]+ = 327.0473) and bis(methylthio)gliotoxin (C15H21N2O4S2; [M+H]+ = 357.0928) with a window of ± 5.0 ppm. GT and bmGT were detected in the A. fumigatus positive control strain Af293. (PDF) [file pgen.1009965.s004.pdf]

## Slide 1
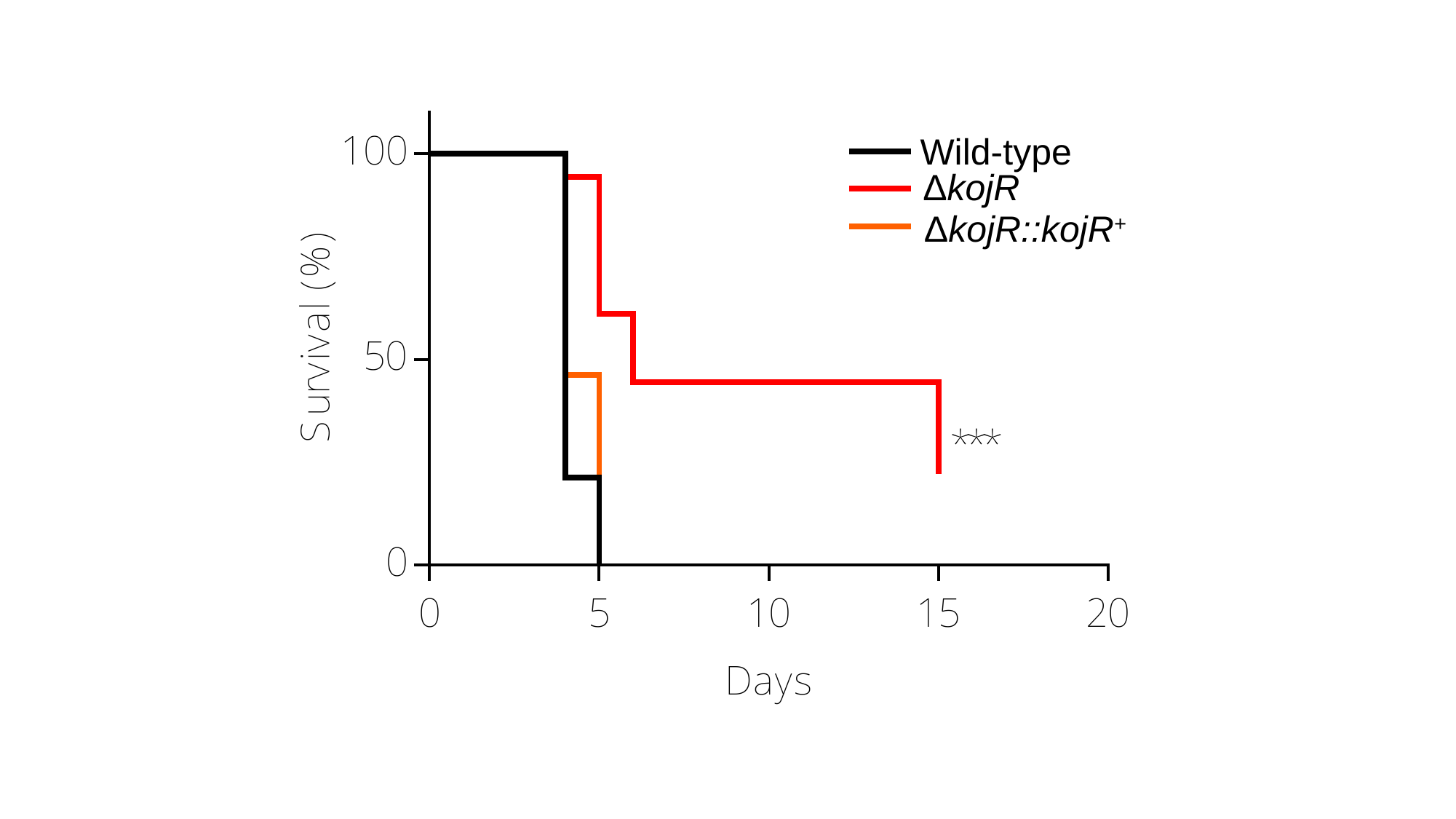

Wild-type
ΔkojR
ΔkojR::kojR+

Supplement: S5 Fig — (PPTX) [file pgen.1009965.s005.pptx]
