# Supplementary material for: Regulation of gliotoxin biosynthesis and protection in Aspergillus species
Source: PLoS Genet. 2022 Jan 18;18(1):e1009965. doi: 10.1371/journal.pgen.1009965 (PMC8797188; doi:10.1371/journal.pgen.1009965)
Supplement: S2 Fig — (PPTX) [file pgen.1009965.s002.pptx]

## Slide 1
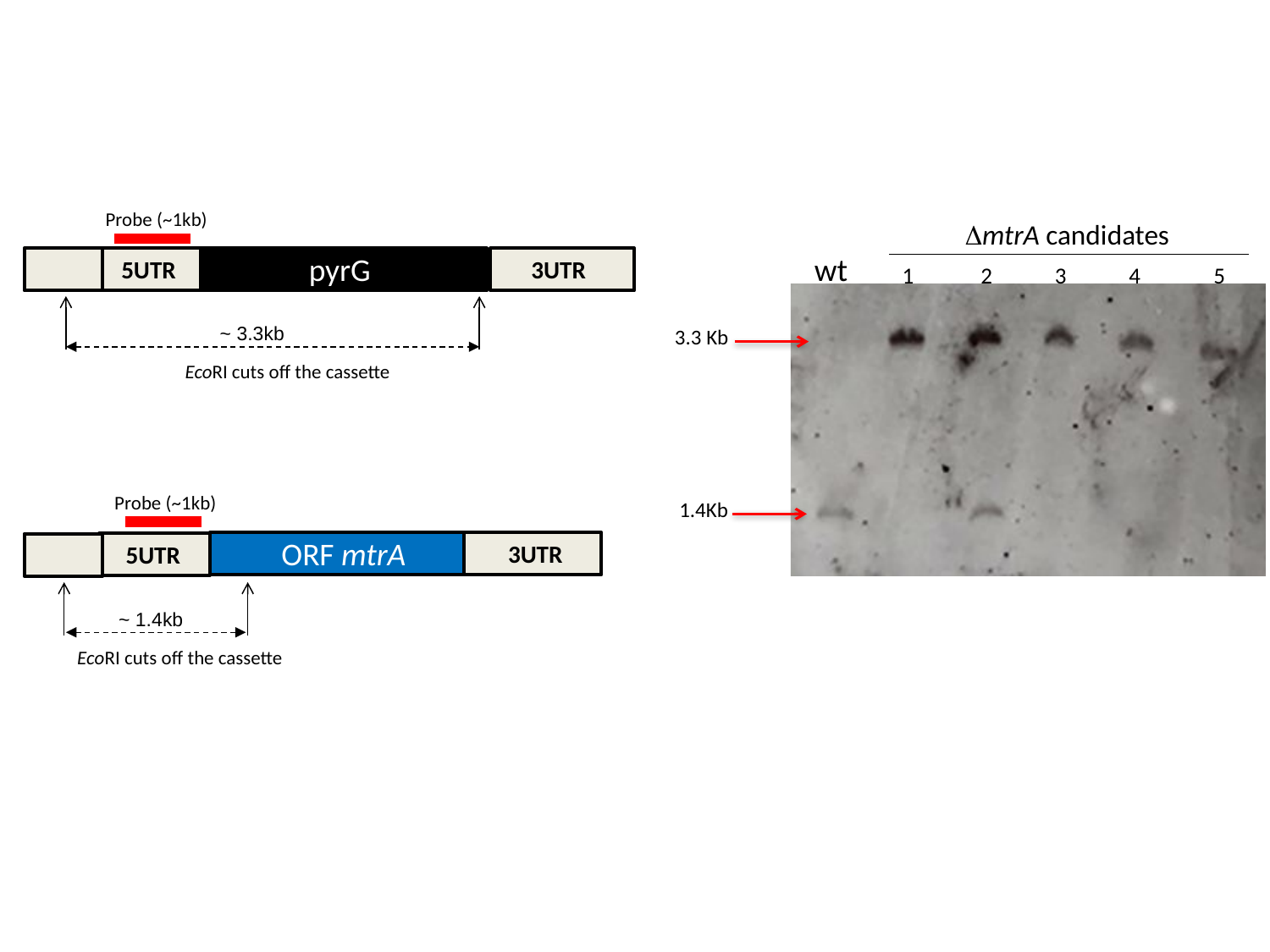

Probe (~1kb)
ΔmtrA candidates
pyrG
wt
5UTR
3UTR
1
3
4
2
5
~ 3.3kb
 3.3 Kb
EcoRI cuts off the cassette
Probe (~1kb)
1.4Kb
ORF mtrA
3UTR
5UTR
~ 1.4kb
EcoRI cuts off the cassette
