# Supplementary material for: Regulation of gliotoxin biosynthesis and protection in Aspergillus species
Source: PLoS Genet. 2022 Jan 18;18(1):e1009965. doi: 10.1371/journal.pgen.1009965 (PMC8797188; doi:10.1371/journal.pgen.1009965)
Supplement: S6 Table — (DOCX) [file pgen.1009965.s011.docx]

**S6 Table - Strains used in this work.**

| **Strain** | **Genotype** | | **Reference** |
| --- | --- | --- | --- |
| ***A. fumigatus*** |  | |  |
| CEA17 | | Δ*akuB pyrG^+^* | [1] |
| CEA17 pyrG^-^ | | Δ*akuB pyrG^-^* | [1] |
| Δ*rglT* | | *MAT1-1*; *delta* *rglT::hph* | [2] |
| Δ*rglT*::*rglT* | | *MAT1-1*; *delta rglT; delta rglT*::*rglT* | [3] |
| Δ*gliT* | | *delta gliT::ptrA* | [4] |
| Δ*kojR* | | *MAT1-1*; *delta* *kojR::hph* | [2] |
| Δ*kojR*::*kojR* | | *MAT1-1*; *delta kojR; delta kojR*::*kojR* | This study |
| Δ*mtrA* | | *delta ku80, pyrG-, mtrA::pyrG+* | This study |
| ***A. nidulans*** | |  |  |
| TN02a3 | | *pyrG89; argB2 delta-nkuA::argB; pyroA4; veA1* | [5] |
| ΔAN1368 (Δ*rglT*) | | *delta AN1368::pyrG; argB2 delta-nkuA::argB; pyroA4; veA1* |  |
| ΔAN1368::AN1368 | | *delta AN1368; delta AN1368::AN1368; argB2 delta-nkuA::argB; veA1* | [3] |
| Δ*kojR* | | *delta kojR::pyrG; argB2 delta-nkuA::argB; pyroA4; veA1* | This study |
| Δ*mtrA* | | *delta mtrA::pyrG; argB2 delta-nkuA::argB; pyroA4; veA1* | This study |
| ***A. oryzae*** | |  |  |
| Wild type | | *delta ku70, pyrG+* | [6, 7,8] |
| Δ*kojR* | | *delta ku70, pyrG-, kojR::pyrG+* |  |
| Δ*rglT* | | *delta ku70, pyrG-, rglT::pyrG+* |  |

**References**

1. Da Silva Ferreira ME, Kress MR, Savoldi M, Goldman MH, Härtl A, Heinekamp T, Brakhage AA, et al. The akuB(KU80) mutant deficient for nonhomologous end joining is a powerful tool for analysing pathogenicity in A*spergillus fumigatus*. Eukaryot Cell. 2006; 5:207-211.
2. Furukawa T, van Rhijn N, Fraczek M, Gsaller F, Davies E, Carr P, et al. The negative cofactor 2 complex is a key regulator of drug resistance in Aspergillus fumigatus. Nat Commun. 2020;11. doi:10.1038/s41467-019-14191-1
3. Ries LNA, Pardeshi L, Dong Z, Tan K, Steenwyk JL, Colabardini AC, et al. The Aspergillus fumigatus transcription factor RglT is important for gliotoxin biosynthesis and self-protection, and virulence. PLoS Pathog. 2020; Jul 15;16(7):e1008645. doi: 10.1371/journal.ppat.1008645.
4. Schrettl M, Carberry S, Kavanagh K, Haas H, Jones GW, O’Brien J, et al. Self-protection against gliotoxin-a component of the gliotoxin biosynthetic cluster, gliT, completely protects Aspergillus fumigatus against exogenous gliotoxin. PLoS Pathog. 2010;6. doi:10.1371/journal.ppat.1000952
5. Hoffmann B, Eckert SE, Krappmann S, Braus GH. Sexual diploids of Aspergillus nidulans do not form by random fusion of nuclei in the heterokaryon. Genetics. 2001;157: 141–147.
6. Jin, F., Nishida, M., Hara, S., Koyama, Y. Identification and characterization of a putative basic helix-loop-helix transcription factor involved in the early stage of conidiophore development in Aspergillus oryzae. Fungal Genet. Biol. 2011; 48, 1108–1115*.*
7. Ogawa, M., Kobayashi, T., Koyama, Y. ManR, a novel Zn(II)_2_Cys_6_ transcriptional activator, controls the β-mannan utilization system in *Aspergillus oryzae*. Fungal Genet. Biol. 2012; 49, 987–995.
8. Tanaka, M., Yoshimura, M., Ogawa, M., Koyama, Y., Shintani, T., Gomi, K. The C_2_H_2_-type transcription factor, FlbC, is involved in the transcriptional regulation of *Aspergillus oryzae* glucoamylase and protease genes specifically expressed in solid-state culture. Appl. Microbiol. Biotechnol. 2016; 100, 5859–5868.
