# Supplementary material for: Regulation of gliotoxin biosynthesis and protection in Aspergillus species
Source: PLoS Genet. 2022 Jan 18;18(1):e1009965. doi: 10.1371/journal.pgen.1009965 (PMC8797188; doi:10.1371/journal.pgen.1009965)
Supplement: S7 Table — (DOCX) [file pgen.1009965.s012.docx]

**S7 Table - List of primers used in this work.**

| Primer | Sequence 5’- 3’ |
| --- | --- |
|  |  |
| Afu1g10910 (tubA) sybr fw | ATATGTTCCTCGTGCCGTTC |
| Afu1g10910 (tubA) sybr rv | GAGAGAGTGGGTGACCTGGA |
| Afu5g06800 sybr fw | ACCTTGCTTGCAATCGACAG |
| Afu5g06800 sybr rv | TGCAGCAGAATGAGCAGGTAG |
| Afu8g07360 sybr fw | TTCGTGCACAGTTGGTTCTG |
| Afu8g07360 sybr rv | TTAATGAACGCGTGCGCATG |
| Afu5g03540 sybr fw | AAGTGTACACGCTCAACACG |
| Afu5g03540 sybr rv | TTCAGCACGATGTTGTACGC |
| Afu3g01750 sybr fw | ACACTCAGTCGCTACAACTACG |
| Afu3g01750 sybr rv | AACGCCGCAAAGAAGAGAAC |
| Afu6g10310 sybr fw | ATGCTGCGTATGCCAATGTC |
| Afu6g10310 sybr rv | TGCCATTGCACGCTATC |
| Afu1g10390 sybr fw | TTGGCGTGGCATTGTTGAAC |
| Afu1g10390 sybr rv | TCTGTGAAAGAGCGGATTCG |
| Afu6g12780 sybr fw | TTGCGGATAAGATGCATGGC |
| Afu6g12780 sybr rv | AACGCCATGTCAGGTTTGTC |
| Afu8g04630 sybr fw | TTGCCTTTGTTGCTGTCGTC |
| Afu8g04630 sybr rv | TAATAGTGGCCAGCGTGAACC |
| Afu5g01350 sybr fw | TGTTTATCGGTCCGTTTGCG |
| Afu5g01350 sybr rv | AGCACAAATCCCAGCAACAC |
| Afu7g065570 sybr fw | TTATTGCTGCGTTGGTGGTC |
| Afu7g065570 sybr rv | AGGAGGATCACGATCGCAATC |
| An7879 sybr fw | AAAGCAGCACTTGACCGTTG |
| An7879 sybr rv | TTTGCAGCAATCGTCGAACG |
| An1472 sybr fw | TGCCGTTATCGCTGTCATTC |
| An1472 sybr rv | ATTGTGGCAAGTGTGAACCC |
| An3717 sybr fw | ACAACAAATGGGCGAGGTTG |
| An3717 sybr rv | TTGCATACCCCGGCATTTTC |
| Gprm sybr fw | GGCGTATCTTTTCGATTCGG |
| Gprm sybr rv | GCTGTTTGTCGTCGAATCGG |
| An8083 sybr fw | TTGTGCTGGCGCTTTGTTTC |
| An8083 sybr rv | ACCTCATCTTGCTTCATGGCA |
| An6963 sybr fw | TGGCACTTTCGAAGAGTGTG |
| An6963 sybr rv | AACGCCTTTCATGCTGGTTC |
| An7190 sybr fw | TGCGTTGAAATGCTCATCCG |
| An7190 sybr rv | AGCCAGTGACACTTTTCGTC |
| An9141 sybr fw | TGACTCGCAAAGGCATCTTC |
| An9141 sybr rv | TGGGAAGCTAGCGCAAATTG |
| An10876 sybr fw | TGTCGCTATGCTGGATCACTC |
| An10876 sybr rv | TGCGATTTCCGGCATAATGG |
| AO090001000037 (gliT) sybr fw | CGCCAAGACTGTCACCATCT |
| AO090001000037 (gliT) sybr rv | TTCGTAGTGCCATCGTCCAG |
| AO090009000281 (tuba) sybr fw | ACCCTGAAACTGGCTTCTCC |
| AO090009000281 (tuba) sybr rv | GTGCAAAGCCCACCATGAAG |
| AO090023000021 (gtmA) sybr fw | TGGAAAGATGCTGCTGGAGT |
| AO090023000021 (gtmA) sybr rv | CATCTTGCTACCGGGGTTGG |
| Afu_gliM sybr fw | CGATCTGTACCCCAACGAGT |
| Afu_gliM sybr rv  Afu_gliA sybr fw | CCCAGGGTCTTCTGGAACTT  TTTGCGATCAACGAACTCTG |
| Afu_gliA sybr rv | CCCTTGACGGACTGGAAGTA |
| Afu_gliH sybr fw | TTGCTGTCCTACCACGACAC |
| Afu_gliH sybr rv | CTTCTTCCGTTCCCGAATCT |
| Afu_gliG sybr fw | CTGATGTACATTGCCGATGC |
| Afu_gliG sybr rv | TTGTTTTGGGCAACTTCTCC |
| Afu_gliP sybr fw | CACAAGAGATGCGCCAGATA |
| Afu_gliP sybr rv | ATCATCGCCTCCATATCAGC |
| Afu_gliK sybr fw | CTCACGGCATACAGCGACTA |
| Afu_gliK sybr rv | ATAATCCAACCGAGCCACTG |
| Afu_gliC sybr fw | TTGTTTGGAGAGGTCCTTGG |
| Afu_gliC sybr rv | CCTTTGTTTTCGAGGCTCTG |
| Afu_gliN sybr fw | GTTCCACGTCCAGTCCATCT |
| Afu_gliN sybr rv | AGACTGCCTTCGGTGAATTG |
| Afu_gliI sybr fw | CCTTCCACCAGATTCCCTTT |
| Afu_gliI sybr rv | CGCTAGCACAATACCCCTGT |
| Afu_gliJ sybr fw | GTATCCGAGGAGGACGTCAA |
| Afu_gliJ sybr rv | CAACTTCCTCACCCGTTCTC |
| Afu_gliF sybr fw | GGGGGCCGATAATATCAACT |
| Afu_gliF sybr rv | AAGATGGCCAATCCACCATA |
| AFUA_1G05020 fw | CGGTCATCGTCCTTTTGATT |
| AFUA_1G05020 rv | CCTTGGACTTGATGGCAGTT |
| AFUA_3G06530 fw | GTAAGAACCACGGTGCCACT |
| AFUA_3G06530 rv | GGGCTTGTACTCATCGGTGT |
| AFUA_1G10820 fw | CGAGGTGTATGTCGATGTGC |
| AFUA_1G10820 rv | CCTCACGGGCAAATCATAGT |
| AFUA_3G06540 fw | TCCATCACTTCCCAGAAACC |
| AFUA_3G06540 rv | GATCGTCTGTCTCCCAGAGC |
| AFUA_2G15590 fw | AACTCGCAGGAAGAAGGACA |
| AFUA_2G15590 rv | GGGTAGATAGGCGACAACGA |
| AFUA_5G02180 fw | CTGCGCTATTCGTTGTCAAA |
| AFUA_5G02180 rv | TACCCTGCGACTGAGTGTTG |
| AFUA_7G01590 fw | AAGGATTGGGCGAAGAAACT |
| AFUA_7G01590 rv | GTCCAACCACGAGACCAAGT |
| AFUA_4G03950 fw | ATGTCTCCGCTCCTTTTGAA |
| AFUA_4G03950 rv | GAAGCGTTGATGGGGAAGTA |
| AFUA_4G07360 fw | GCCACCTACTTCGGTGACAT |
| AFUA_4G07360 rv | GTTGCGACCATCAACAACAC |
| AFUA_1G10630 fw | CCTTGTCCAGCTCTCCTACG |
| AFUA_1G10630 rv | GGAAGCTCTGGTTGGTGAAG |
| AFUA_2G07620 fw | CCAGAGGCACCAAAGGATAA |
| AFUA_2G07620 rv | TTTCCCAAAGTGACCAGTCC |
| AFUA_8G04340 fw | GGAGAGTCTCTGCGAAGTGC |
| AFUA_8G04340 rv | CTGGCTAGCGAGAACAGCTT |
| AFUA_5G04250 fw | TTGGGGATTCGAAGACTCTG |
| AFUA_5G04250 rv | CTGTTCAAAATCGGCAATGA |
| AFUA_3G13900 fw | GCGTCCAACTCCACCTACAT |
| AFUA_3G13900 rv | GCCTTGTTCAAATCCAGCTC |
| AFUA_5G06610 fw | CTTCTCTGGTGCGAAAGCTC |
| AFUA_5G06610 rv | CGACTTCGAAGGACCGTAAG |
| AFUA_2G15650 fw | GGACAATGAGAAGCCTCAGC |
| AFUA_2G15650 rv | AGAGTCACCCCCTTGGAGAT |
| Aori sB sybr fw | GACTCGGATGAAGGATTCCA |
| Aori sB sybr rv | TGGTCTCTGAATCGTGCTTG |
| Ani sB syb fw | CCGTGATCGTTCTCCTGATT |
| Ani sB syb rv | CGCGAAAAAGATCCAGTAGC |
| AfukojR complemented 5UTR pRS fw | GTAACGCCAGGGTTTTCCCAGTCACGACGATGTAATGGCGACGGTCTTGT |
| AfukojR complemented 3UTR pRS rv | GGATAACAATTTCACACAGGAAACAGCCTTGTTTTCATTTCCCTCGCG |
| AfukojR 5UTR ext fw | ACAATGTAGTACTTGGTTGTGCA |
| AfukojR 3UTR ext rv | CCGCCTTCCAGTACTACAGTC |
| AfuRglT_5UTR RV_ptrA | CAATTGCCCGTCTGTCAGATCGTTGAATGATCTGGATGGAGA |
| AfuRglT_3UTR FW_prtA | CGGCTCATCGTCACCCCATGATAGGCGATCTGTTTGTTTACCTACGTG |
| AniKojR_5UTR_pyro_RV | GACCCAACAACCATGATACCACCTAACCTATCCACAGGAGGACT |
| AniKojR_3UTR_pyro_FW | CTGTCGATCATGTGGATGCTCAAGTTGGTCAAGAGTTGTACTA |
| prtA FW | GATCTGACAGACGGGCAATTG |
| prtA RV | CTATCATGGGGTGACGATGAGCCG |
| AN4118 (kojR)_pRS426_5UTR_fw | GTAACGCCAGGGTTTTCCCAGTCACGACGAGCAATAGGGGCAATAAATGGGC |
| AN4118_pRS426_3UTR_rv | GCGGATAACAATTTCACACAGGAAACAGCTGTTCTAGCCCTACTTCCGTGAG |
| AN4118_5UTR_pyrG_rv | CAGTGCCTCCTCTCAGACAGAATCCTAACCTATCCACAGGAGGACT |
| AN4118_3UTR_pyrG_fw | GAGCATTGTTTGAGGCGAATTCCAAGTTGGTCAAGAGTTGTACTA |
| AN4118 ext_5UTR_fw | CACATTTCCATTCTCCGCCAGTG |
| AN3717_pRS426_5UTR FW | GTAACGCCAGGGTTTTCCCAGTCACGACGCTTCTACTCCTGATTCATGGCTGA |
| AN3717_pRS426_3UTR RV | GCGGATAACAATTTCACACAGGAAACAGCAAATGAAGCATGTACGACGTTCTC |
| AN3717_5UTR_pyrG_RV | CAGTGCCTCCTCTCAGACAGAATCATTTCGCCGGTAAGACACTAGAA |
| AN3717_3UTR_pyrG_FW | GAGCATTGTTTGAGGCGAATTCGGTTCTCGGAGGGAAAAGCTGTTT |
| AN3717 ext_homologia | TGACGATTCGTGTCAACCCTGAAT |
| Afu6g12780_pRS426_5UTR FW | GTAACGCCAGGGTTTTCCCAGTCACGACGTGTTCACTTTGTCGGCAAGCGATG |
| Afu6g12780_pRS426_3UTR RV | GCGGATAACAATTTCACACAGGAAACAGCGAGATCACCAGTCGGCTTGAGATG |
| Afu6g12780_5UTR_pyrG_RV | AGTGCCTCCTCTCAGACAGAATCGTGATGATTTGAAAGGCTTCTGAGC |
| Afu6g12780_3UTR_pyrG_FW | GAGCATTGTTTGAGGCGAATTCTGCACGCTGTGCGCTTCTTAAGCA |
| Afu6g12780 ext_homologia | TGCAGTAGGAGTTCACCAAAGTTG |
