# Supplementary material for: Regulation of gliotoxin biosynthesis and protection in Aspergillus species
Source: PLoS Genet. 2022 Jan 18;18(1):e1009965. doi: 10.1371/journal.pgen.1009965 (PMC8797188; doi:10.1371/journal.pgen.1009965)
Supplement: Attachment — Submitted filename: Answers to the Reviewers.docx [file pgen.1009965.s013.docx]

Answers to the Reviewers

Reviewer #1:

Gliotoxin is a well-characterized Aspergillus fumigatus secondary metabolites, and is known to impair several aspects of the human immune response. The authors have previously identified the transcription factor RglT, which is important for GT biosynthesis and self-protection, and found that RglT is also present in non-GT-producing fungus A. nidulans. In this manuscript, the authors investigated a possible regulation mechanism for GT protection in three Aspergillus species (Af, An and Ao) by comparative transcriptome profiling (Af and An) and screening the Af TF factor deletion set. They identified a novel TF KojR involved in GT protection. However, there is no clear mechanism explaining the role of KojR in GT protection in the three species examined.

Answer: We thank the reviewer for the comments and suggestions. In this new revised version we have provided novel information about *A. fumigatus* KojR showing that this transcription is important for inorganic sulfur assimilation when *Aspergillus spp* are challenged with gliotoxin, and also virulence.

Major comments

1.The authors aimed to identify the novel components required for GT protection by comparing the transcriptional response to exogenous GT in Af and An. They found that the transcriptional response to GT is different between the species, although the response of 55 genes was shared. However, the shared gene list failed to provide any clear explanation for GT resistance.

Answer: Now we have added additional evidence that one of the genes regulated by *A. fumigatus* and *A. nidulans* Rglt, *mtrA* encoding a methyltransferase, is important for gliotoxin protection (lines 300 to 318): “**A novel methyltransferase (MtrA) is important for GT self-defense in *Aspergilli*.** Among the genes dependent on *Aspergilli* RglT, we identified a methyltransferase (named MtrA, AN3717/AFUA_6G1278). (Figure 2C and Supplementary Table S4). We deleted this gene in both *A. nidulans* and *A. fumigatus* and tested the growth of two independent transformants for each deletion in the presence of GT (Supplementary Figure S3 and Figure 3A). *A. fumigatus* Δ*mtrA* deletion strains showed about 20 % growth reduction compared to the corresponding wild-type while *A. nidulans* Δ*mtrA* deletion strains presented about 60 % growth reduction (Figure 3A). Figure 3B shows the taxonomic distribution of MtrA, RglT, GliT, and GT biosynthetic gene cluster (BGC) homologs across Eurotiomycetes and Sordariomycetes. Phylogenetically informed model testing of the distributions of RglT and MtrA suggests that the pattern of occurrence of RglT is statistically dependent on the distribution of MtrA but not vice-versa. This analysis suggest an evolutionary scenario in which the MtrA-based resistance mechanism is ancestral and the evolutionary recruitment of RglT regulation of GliT occurred subsequently.

These results strongly indicate that our approach to compare the transcriptomics of *A. fumigatus* and *A. nidulans* Δ*rglT* mutants was efficient to identify novel genes involved in GT-protection.”

.

2.In the second part of this manuscript , the authors used the Af TF deletion library to screen for GT sensitivity. They identified a few TF mutants that are sensitive to GT and further characterized the KojR deletion strain. However, there is no specific mechanism identified for KojR’s mode of action. Many key questions remained unaddressed, including how KojR and RglT work together to regulate the expression of gliT. There is no experimental evidence to verify that the detoxification of KojR is dependent on the expression of gliT or other oxidoreductase encoding genes.

Answer: We added new data showing that KojR is important for the regulation of *gliJ* and genes involved in the inorganic sulfur assimilation and transsulfuration. Although RglT regulates directly *gliT* mRNA accumulation, our new data suggest that there is a global reduction of the mRNA accumulation of 20 to 30 % *gli* genes in the Δ*kojR* mutant (except *gliZ* that has 10 % higher mRNA accumulation in the Δ*kojR* mutant) upon gliotoxin exposure, and *gliJ* that is completely downregulated in the Δ*kojR* mutant.

Minor comments

1.Page 14, line 421-423: The results show the KojR induces the expression of gliT, but the expression of gliT in AfKojR deletion strain is actually higher in gliotoxin condition.

Answer: The *gliT* mRNA accumulation is 20 % lower in the *A. fumigatus* Δ*kojR* than in the wild-type.

2.Please write the RT-qPCR in the same way.

Answer: This was now modified to RT-qPCR along the manuscript.

3.Fig. 4: minimal medium supplemented with the compounds (A-H), please remove the MM (E-H).

Answer: This was now modified.

4.Fig. 6C: one strain name is missing.

Answer:This was now added to the Figure.

5.The kojR complemental strain needs to be included in Fig. 6C.

Answer: We do not have na *A. fumigatus* complementary strain for Δ*kojR*. Instead of it, we added two independent deletion strains.

6.Fig. 6D: kojR deletion strain in A. oryzae increased sensitivity compared to that in A. fumigatus. Please explain or discuss it.

Answer: We cannot explain the increased gliotoxin sensitivity of the *A. oryzae* Δ*kojR* mutant. We have added a sentence discussing it (lines 418 to 426): “Similarly to *A. fumigatus*, deletion of *kojR* significantly increased sensitivity to exogenously added GT in both *A. nidulans* and *A. oryzae* (Figures 7B, 7C and 7D). In contrast to the deletion of *rglT*, which makes all three species sensitive to GT, deletion of *kojR* conferred some resistance to exogenous 30 or 10 µg/ml GT, respectively, in *A. fumigatus* and *A. nidulans* (Figures 7B and 7C) but was completely sensitive to 30 µg/ml GT in *A. oryzae* (Figure D). We cannot explain this increased sensitivity to GT in *A. oryzae* Δ*kojR*, but these results suggest that RglT functions downstream of KojR and/or that RglT is regulated by KojR in these conditions.”

7. Page 39, Line 1217: The gliotoxin concentration should be 30 μg/ml and in A. nidulans is 10 μg/ml.

Answer: This was now modified.

8.Page 40, line 1224-1234: Is the cultural time 20 h or 21 h?

Answer: The cultural time was 20 hs and this was now stated in all the sections of the manuscript.

Reviewer #2:

This manuscript describes a global transcriptional analysis to analyze common responses to gliotoxin stress in multiple Aspergillus species, coupled with a screen of the currently available A. fumigatus transcription factor deletion library to identify novel transcription factors involved in gliotoxin protection. The authors report that the distantly related organisms, A. fumigatus and A. nidulans, display differential responses to exogenous gliotoxin stress but are able to identify a core set of homologous genes that similarly regulated between the species. Regulation of these genes is subsequently confirmed by qRT-PCR. Given that a subset of these genes are regulated through a mechanism independent of the known regulator, RglT, the authors then screened the transcription factor deletion library for additional regulators. The authors identified 15 additional TFs that are required for normal growth rates in the presence of GT and chose on TF (KojR) for further analysis based on the finding that this TF mutant produces significantly less GT and BmGT. Known for it’s role in kojic acid biosynthesis in A. oryzae, deletion of the KojR ortholog from disparate species also revealed that this TF plays a conserved role in GT protection. Further transcriptional studies show that RglT and KojR crosstalk at the transcriptional level and that both RglT and KojR are required for transcriptional regulation of the gliotoxin genes, gliT and gtmA, in response to GT stress. Overall, this is an interesting study that is well written. The conclusions are largely supported by the data and statistical tests have been appropriately applied.

Answer: We thank the reviewer for the comments and suggestions.

1. Line 276-279: It is very confusing that GliT (a major GT-protection component) is described in the RNAseq data as being Rglt-independent and is differently regulated in response to GT in both A. fumigatus (down-regulated) and A. nidulans (up-regulated). However, in the qRT-PCR data presented in Figure 7C, gliT is not only described as being upregulated in both A. nidulans and A. fumigatus but also as being rglT-dependent. Transcriptional changes are not always confirmed by qRT-PCR, obviously, but the conflicting data regarding rglT-dependence needs to be explained.

Answer: We apologize about the lack of clarity. This is a duplication of GliT and it is not known if it is involved in gliotoxin protection. We changed the word “homologue” by “paralogue” in the corresponding sentence (lines 283 to 286): “Another RglT-independent gene is the GliT paralogue AN6963/AFUA_5G03540; interestingly, this gene is upregulated in *A. nidulans* and downregulated in *A. fumigatus* upon GT exposure (Supplementary Table S4).”

2. As written, the manuscript is really in two parts. The first describing the RNAseq dataset defining conserved and unique transcriptional changes in response to GT stress and the second identifying novel TFs regulating GT protection. In the first part of the manuscript, the authors have identified what appear to be conserved transcriptional responses that are independent of the major GT stress regulator, RglT. It is perfectly reasonable then to screen the TF deletion library for novel regulators. It seems extremely important that, since the authors have identified a novel TF important for GT protection, studies should be performed to see if this novel TF is required for differential expression of any of those conserved genes. This logical next step would add needed mechanistic insights and would bring together the two unconnected parts of the study. (Lines 280-285).

Answer: We thank the reviewer for this excelent suggestion. We have added an extensive study of genes involved in the gliotoxin production and protection. Our results indicate that kojR regulates *gliJ* and it is important for the regulation of inorganic sulfur assimilation and transsulfuration upon gliotoxin stress.

3. The stresses generated in both species by addition of exogenous GT could be very different depending on the GT MIC for each. The concentration of GT was chosen based on A. fumigatus susceptibility. What is the GT MIC for A. nidulans? If it is much lower than A. fumigatus, the level of stress induced (and therefore the specific genes that respond transcriptionally and the magnitude of that response) may not be directly comparable as the authors have done here. This is largely OK, as the authors focus on the genes in common for further analyses. However, the conclusion in Lines 286-288 that the “transcriptional response of A. fumigatus and A. nidulans to GT is very different” must be taken with this caveat. This should be clearly stated in the text.

Answer: We thank the reviewer for this comment. We have added to the text the *A. nidulans* MIC (lines 231 to 233): “Next, we performed RNA-seq for the *A. nidulans* WT (the GT MIC for *A. nidulans* is 30 µg/ml) and ∆*rglT* strains in the same conditions (5 µg/ml GT for 3 h) as described above and identified DEGs.”

In addition, we acknowledged that *A. nidulans* is more susceptible to gliotoxin and that and the level of stress induced (and therefore the specific genes that respond transcriptionally and the magnitude of that response) may not be directly comparable (lines 293 to 298): “Taken together, our results suggest that the transcriptional responses of *A. fumigatus* and *A. nidulans* to GT are very different, although we were able to observe 54 homologs whose transcriptional response was similar in both species. However, we note that we used an equal exposure to GT (5 µg/ml GT for 3 hours) for both species but *A. nidulans* shows greater susceptibility to GT than *A. fumigatus*.”

4. The description of data presented in Fig 7A and 7B are reversed in the text. Please correct. (Lines 398-406)

Answer: This was now fixed.
