# Supplementary material for: Regulation of gliotoxin biosynthesis and protection in Aspergillus species
Source: PLoS Genet. 2022 Jan 18;18(1):e1009965. doi: 10.1371/journal.pgen.1009965 (PMC8797188; doi:10.1371/journal.pgen.1009965)
Supplement: Attachment — Submitted filename: Comments for de Castro et al..docx [file pgen.1009965.s014.docx]

**Comments for de Castro et al.**

***mtrA* nomenclature:** The *∆mtrA1* and *∆mtrA2* nomenclature (Figure 3) gives the impression that these are deletions in separate genes, i.e. paralogs of *mtrA*. To indicate independent mutants in the same gene, a better system would be (*∆mtrA-1*, *∆mtrA-2* or ∆mtrA #1, *mtrA* #2).

**Figure 6**: What exactly is being plotted in the heatmap (Figure 6c)? Is this a relative fold change between WT and the respective mutants? Some specific points of confusion for me include:

- 5G14390 and 6G09870 are both bright yellow for GT whereas WT is black. This would suggest that there is considerably more GT in those two mutants relative to WT. However, the levels of GT plotted in 6a are identical (not statistically different) for each of those strains.
- 6G09930 is a less bright yellow (mustard?) for GT, indicating that it produces less toxin than either 55G14390 or 6G09870. According to Figure 6a, however, the mutant produces more GT!
- 8G07280 displays a yellow shade (same as 6G09930), but in Figure 6a the mutant produces the same amount (if not less) than WT.

So, is there something wrong with this figure, or am I missing something?

**Figure 7E**: The bottom row of plates in 7E (i.e. the row with no visible fungus) is not described in the results or figure legend. Please do so.

**Line 682**: *“These results suggest that KojR dependent regulation of gliT differs between GT-producing and non-producing Aspergillus species and these results are in agreement with our A. fumigatus/A. nidulans RNA-seq dataset.”*

- This passage is one of many in the manuscript relating to the idea that that mechanisms of GT synthesis and resistance vary between *Aspergillus* species. However, the authors should discuss the possibility that there may be just as much variability within species, i.e. strain heterogeneity. Indeed, the authors mention that *∆rgdA* mutants in Af293 and CEA17 display qualitatively distinct phenotypes with respect to GT production. The authors are no doubt aware (and have even reported on!) such intra-species variability with respect to other pathways/stress responses. This should be a part of this discussion.

**Minor edits**

**Line 372**: *“…produces a similar of bmGT…”*

- Missing a word.

**Line 421**: *“In contrast to the deletion of rglT, which makes all three species sensitive to GT,* *deletion of kojR conferred some resistance to exogenous 30 or 10 µg/ml GT, respectively…”*

- The phrase “conferred some resistance” makes it sound like the *∆kojR* mutants are more resistant than WT. I think the authors mean to say that the *∆kojR* mutants, while still hypersensitive to GT compared to WT, are more resistant than the *∆rglT* mutants.

**Line 548**: *“…demonstrated a significantly reduced inflammation score (Figure 11C) and hyphal invasion of the tissue (Figure 11D) relative to the WT and ΔkojR::kojR strains.”*

- I think the authors mean immune cell invasion, not “hyphal” invasion.
