# Supplementary material for: Regulation of gliotoxin biosynthesis and protection in Aspergillus species
Source: PLoS Genet. 2022 Jan 18;18(1):e1009965. doi: 10.1371/journal.pgen.1009965 (PMC8797188; doi:10.1371/journal.pgen.1009965)
Supplement: Attachment — Submitted filename: Answers to the Reviewers.docx [file pgen.1009965.s015.docx]

Answers to the Reviewers

Reviewer #1:

The article presented here, further provides within the mechanisms behind the regulation of gliotoxin in the human pathogenic fungus Aspergillus fumigatus. Furthermore, the article presents a new transcription factor involved in the defense during the exposure of this relevant mycotoxin. Gliotoxin toxicity is very unspecific, affecting protein folding, oxidative stress, methylation and probably chromatin structures and global gene expression. Therefore, it is not surprising that a large number of transcription factors are directly and indirectly influenced by the exogenous exposure of this toxin. However, the strength of the article is to include in the study also gliotoxin non-producing fungi, pointing to interspecies defense mechanisms.

Overall, the article was well presented, even if the amount of the data reported is massive. This obviously makes reading and understanding is not easy. Nevertheless, the efforts made by the authors are appreciable. Since the authors have already addressed an extensive revision process, I have no further experiments to ask, but I would appreciate some changes in the text.

Answer: We thank the reviewer for the comments and suggestions.

Lines 74-75: The authors wrote: “KojR regulates the expression of another TF, an oxidoreductase, …”. This sentence is confusing, it seems like KojR is an oxidoreductase. Please change.

Answer: The sentence was changed to (lines 74 to 75): ”KojR regulates the expression of genes important for gliotoxin biosynthesis and protection, and sulfur metabolism.”

Line 158: Please remove “and is distantly related to A. fumigatus”; it is redundant with line 165.

Answer: The sentence was changed to (lines 156 to 158): “Interestingly, RglT and GliT were shown to have similar roles in GT protection in *A. nidulans*, a fungus that does not produce GT (Ries *et al*., 2020).”

Lines 172-175. This paragraph is confusing. I would suggest: We found that one of these TFs is a KojR hortolog, previously reported as regulator of the kojic acid production gene cluster in A. oryzae, is important as well for A. nidulans and A. oryzae GT protection and involved in A. fumigatus virulence, GT self-protection and GT and bmGT biosynthesis. Or something similar…

Answer: The sentence was changed to (lines 172 to 175): “We found that one of these TFs is a KojR ortholog, previously reported as regulator of the kojic acid production gene cluster in *A. oryzae*, is important as well for *A. nidulans* and *A. oryzae* GT protection and involved in *A. fumigatus* virulence, GT self-protection and GT and bmGT biosynthesis.”

Lines 272-279: Is it necessary to list all those genes here? A potential reader can see them on the figure… The same for lines 327-334.

Answer: We thank the reviewer for the suggestion. Since the genes described in lines 272 to 279 are dependent or not on RglT, we would prefer to keep them in the text. However, as requested by the reviewer, we have removed the genes from lines 327-334.

Lines 351-352: Why is it interesting? I could not get it.

Answer: The reviewer is right and the word “Interestingly” was removed. The sentence now is (lines 347 to 348): ‘The ∆AFUA_8G07360 mutant is resistant to diamide (Figure 5D).”

Line 633: according to your results reported in figure 6 there is still a minor production of gliotoxin in the Delta-kojR mutant strain. Am I wrong?

Answer: The reviewer is right. The sentence was changed to (lines 626 to 628): “In contrast, the deletion of *kojR* was the only mutant strain where reduced amounts of GT and bmGT was observed, suggesting that this TF is important for GT metabolism.”

Line 715-717: this statement is very speculative. If there are no data supporting that, please remove it.

Answer: The sentence was removed.

Lines 739-741: how this work may lead to the development of anti-fungal strategies? Sounds like a "pret-a-porter" closing remark.

Answer: The sentence was changed to (lines 734 to 736): “More importantly, the identification of a conserved transcriptional response to exogenous GT in different *Aspergillus* species and isolates provides a basis for future studies which will further decipher these protection pathways.”

Reviewer #2:

This revised manuscript is very nice. My comments are minor:

Answer: Thank you very much for your comments and suggestions.

1) The sentence in lines 420-422 needs to be re-written because it seems to imply that deletion of kojR results in resistance to GT. For example, the sentence could be changed to: “In contrast to deletion of rglT, which made all three species fully sensitive to GT, deletion of kojR resulted in only partial sensitivity to GT”.

Answer: The sentence was changed to (lines 416 to 418): “In contrast to deletion of *rglT*, which made all three species fully sensitive to GT, deletion of *kojR* resulted in only partial sensitivity to GT (Figures 7B, 7C and 7D).”

2)Fig. 8E is mentioned before Fig. 8D in the text. These two figures should be switched.

Answer: The figures were switched.

Reviewer #3:

In this manuscript, de Castro and colleagues utilize transcriptomics and a knockout library screen to identify transcription factors with a previously unidentified role GT production and protection in Aspergillus. Overall, the data are thorough and well-presented, and the manuscript itself is well-written. While I do not have any experimental suggestions or concerns, I do have a few editorial notes that should be considreed to improve clarity or bolster the discussion. These are outlined below and in the attached document.

Answer: We thank the reviewer for the comments and suggestions.

Major comments:

1) mtrA nomenclature: The ∆mtrA1 and ∆mtrA2 nomenclature (Figure 3) gives the impression that these are deletio ns in separate genes, i.e. paralogs of mtrA. To indicate independent mutants in the same gene, a better system would be (∆mtrA-1, ∆mtrA-2 or ∆mtrA #1, mtrA #2).

Answer: We have changed the nomenclature in Figure 3 to Δ*mtrA-1* and Δ*mtrA2*, and also in Figures 7 and 10 for Δ*kojR-1* and Δ*kojR-2*.

2) Figure 6: What exactly is being plotted in the heatmap (Figure 6c)? Is this a relative fold change between WT and the respective mutants? Some specific points of confusion for me include:

- 5G14390 and 6G09870 are both bright yellow for GT whereas WT is black. This would suggest that there is considerably more GT in those two mutants relative to WT. However, the levels of GT plotted in 6a are identical (not statistically different) for each of those strains.

- 6G09930 is a less bright yellow (mustard?) for GT, indicating that it produces less toxin than either 55G14390 or 6G09870. According to Figure 6a, however, the mutant produces more GT!

- 8G07280 displays a yellow shade (same as 6G09930), but in Figure 6a the mutant produces the same amount (if not less) than WT.

So, is there something wrong with this figure, or am I missing something?

Answer: We thank the reviewer for realizing these mistakes in the Figure 6. The results are expressed as absolute values. There was a problem with the order of the mutants in the figure and the corresponding order in the heat map. A new Figure 6 correcting these mistakes was added to the manuscript. This new Figure does not change the results in the manuscript. We apologize about the mistake.

Figure 7E: The bottom row of plates in 7E (i.e. the row with no visible fungus) is not described in the results or figure legend. Please do so.

Answer: We have added to the legend of the Figure 7: “The first and second rows indicate the top and bottom of the plates, respectively.”

Line 682: “These results suggest that KojR dependent regulation of gliT differs between GT-producing and non-producing Aspergillus species and these results are in agreement with our A. fumigatus/A. nidulans RNA-seq dataset.”

- This passage is one of many in the manuscript relating to the idea that that mechanisms of GT synthesis and resistance vary between Aspergillus species. However, the authors should discuss the possibility that there may be just as much variability within species, i.e. strain heterogeneity. Indeed, the authors mention that ∆rgdA mutants in Af293 and CEA17 display qualitatively distinct phenotypes with respect to GT production. The authors are no doubt aware (and have even reported on!) such intra-species variability with respect to other pathways/stress responses. This should be a part of this discussion.

Answer: We thank the reviewer for this important observation. We have modified the last paragraph of the Discussion section aiming to address this observation (lines 726 to 736): “There is extensive heterogeneity in drug susceptibility, nutritional requirements, and virulence across *A. fumigatus* clinical and environmental isolates (Kowalski *et al.*, 2016; Ries *et al*., 2017, 2019; Colabardini *et al*., 2021). It remains to be determined if GT-sensitive mutants in different *Aspergillus spp* isolates will behave accordingly to what was observed in this manuscript using the available isolates. Our work provides new opportunities to understand GT production and protection, and also highlights significant differences in transcriptional responses to extracellular mycotoxins between fungal species. More importantly, the identification of a conserved transcriptional response to exogenous GT in different *Aspergillus* species and isolates provides a basis for future studies which will further decipher these protection pathways.”

Minor edits:

Line 372: “…produces a similar of bmGT…”

- Missing a word.

Answer: The sentence was changed to (line 368): “...and produces a similar amount of bmGT when compared to the WT..”

Line 421: “In contrast to the deletion of rglT, which makes all three species sensitive to GT, deletion of kojR conferred some resistance to exogenous 30 or 10 µg/ml GT, respectively…”

- The phrase “conferred some resistance” makes it sound like the ∆kojR mutants are more resistant than WT. I think the authors mean to say that the ∆kojR mutants, while still hypersensitive to GT compared to WT, are more resistant than the ∆rglT mutants.

Answer: As requested by reviewer #2, the sentence was changed to (lines 420 to 422): “The sentence was changed to (lines 416 to 418): “In contrast to deletion of *rglT*, which made all three species fully sensitive to GT, deletion of *kojR* resulted in only partial sensitivity to GT (Figures 7B, 7C and 7D).”

Line 548: “…demonstrated a significantly reduced inflammation score (Figure 11C) and hyphal invasion of the tissue (Figure 11D) relative to the WT and ΔkojR::kojR strains.”

- I think the authors mean immune cell invasion, not “hyphal” invasion.

Answer: We thank the reviewer for the suggestion. We modified it (lines 542 to 544): “...demonstrated a significantly reduced inflammation score (Figure 11C) and immune cell recruitment to the tissue (Figure 11D) relative to the WT and Δ*kojR*::*kojR* strains.”
